# The Scope of Respiratory Syncytial Virus Infection in a Tertiary Hospital in the Eastern Province of Saudi Arabia and the Change in Seasonal Pattern during and after the COVID-19 Pandemic

**DOI:** 10.3390/medicina58111623

**Published:** 2022-11-10

**Authors:** Khaled R. Alkharsah

**Affiliations:** Department of Microbiology, College of Medicine, Imam Abdulrahman Bin Faisal University (IAU), P.O. Box 1982, Dammam 31441, Saudi Arabia; kalkharsah@iau.edu.sa; Tel.: +966-133331053

**Keywords:** Respiratory syncytial virus, RSV, COVID-19, hospitalization, seasonality

## Abstract

*Background and Objectives*: Respiratory syncytial virus (RSV) is a major cause of morbidity and hospital admission due to respiratory tract infection among infants and young children. The current study aims to describe the prevalence and the seasonal pattern of RSV during the previous seven years. *Materials and Methods:* Clinical data and RSV antigen and PCR test results were collected from patients’ medical records at King Fahd Hospital of the University in the Eastern Province of Saudi Arabia between January 2015 and February 2022. *Results:* The overall percentage of RSV detection was 26.3% (336/1279) among the tested individuals. RSV infection was more common among children below five years and elderly above 60 years of age. Two-thirds of the cases required hospitalization. The average hospital stay due to RSV infection was 6.5 days (range 0–56 days). The rate of hospitalization was higher among infants and younger children and decreased with age (*p*-value < 0.001). RSV infection was more prevalent between August and February and decreased appreciably between March and July. The peak level of infection was during December and January. No RSV infections were reported during the COVID-19 pandemic and the following winter. The cases increased again in August 2021, with an unusual out-of-season peak. *Conclusions:* RSV infection is one of the important causes of morbidity and hospitalization among infants and young children in Saudi Arabia. The seasonal pattern of infection has changed after the COVID-19 pandemic, and the physicians should be aware that infection may happen currently at different times throughout the year.

## 1. Introduction

Respiratory syncytial virus (RSV) is an enveloped RNA virus within the family *Pneumoviridae* and the genus *Orthopneumovirus*. Infection with RSV is very common during early childhood. Nearly all children become infected with RSV before they complete 2 years of life [1]. Nonetheless, infection with RSV does not cause lifelong immunity; therefore, it can happen repeatedly at any age [2]. The virus is highly contagious and transmits between individuals either through large and small aerosol particles or through contaminated fomites [3]. RSV infection shows a seasonal transmission pattern with regional and geographical variability. It occurs between October and May in the Northern Hemisphere, while it tends to follow the rainy season in the Southern Hemisphere and tends to last the whole year in the tropical regions [4].

In immunocompetent children and adults, RSV infection mostly causes mild infection, while in infants and young children, it could develop into lower respiratory tract infection (LRTI) presenting in the form of bronchiolitis and/or pneumonia and may require hospitalization [5]. A higher risk of developing severe RSV infection with complications is associated with premature children and children with comorbidities, such as cystic fibrosis, congenital heart disease, immunodeficiency, and bronchopulmonary dysplasia [4]. Immunocompromised adults and elderly people above 65 years old are also at high risk of developing severe RSV infection [4].

There is currently no approved vaccine or treatment recommended for RSV infection. The only available monoprophylaxis for specific high-risk pediatric children is palivizumab, which is a monoclonal antibody that targets the virus fusion (F) protein [2,6].

In 2019, it was estimated that over 33 million episodes of RSV infections were reported globally in children below 5 years of age [7]. These episodes resulted in more than 3.5 million hospital admissions and about 60 thousand hospital deaths worldwide [7]. RSV is identified as the principal cause of hospitalization in the United States in infants due to upper respiratory tract infection (URTI) [8]. Adults’ infection with RSV leads to an average of 150 thousand hospitalizations with up to 17 thousand deaths annually in the United States, with a cost of up to USD 5 billions [9]. It was estimated that the RSV disease burden in Europe is up to 2.5-fold higher than that in the United States [10]. The annual rate of RSV hospitalization was estimated to be between 40 and 85 per one thousand infants during their first two months of life and decreases to range between seven to twenty per one thousand older infants, with a median length of hospital stay between two to four days [11,12]. RSV was found to be the most common cause of non-influenza respiratory cases in 19 counties from the eastern Mediterranean region between 2016 and 2018, accounting for 35.9% of cases [13].

Several studies from Saudi Arabia described the prevalence of RSV to range between 0.2–54% among multiple age groups during the period between 1991 and 2015 [14,15]. A later study from Jeddah estimated the prevalence of RSV to be 13.4% in 2017 [16]. Nonetheless, all studies are localized to the central, west, and south regions of the country and during a short period of one or two years. The purpose of the current study is to look for the prevalence of RSV infection in the eastern region of the country over a period of more than seven years, spanning the COVID-19 pandemic.

## 2. Materials and Methods

Study Type: Retrospective Study

Study settings: The study was conducted at King Fahd Hospital of the University (KFHU), which is a tertiary hospital serving general population in the Eastern Province of Saudi Arabia. KFHU comprises a total of 754 beds (502 inpatient beds, 56 emergency beds, 77 ICU beds, 32 psychiatry beds, and 87 special auxiliary beds). All data were collected from the patients’ medical records in the hospital. The data search included any medical request for RSV testing during the period from January 2015 till February 2022. Information on demographic data, clinical manifestations, and days of hospitalization was collected. Additionally, risk factors such as preterm delivery, body weight, chronic lung disease, and congenital heart disease were also investigated among RSV-positive patients.

RSV testing: A total of 1279 respiratory samples were screened for RSV infection between January 2015 and February 2022. The respiratory samples were either tested with RSV antigen test (53.1%) or RSV PCR test (46.9%). The RSV antigen test was used from 2015, while the PCR test was introduced in 2017 and was more employed over time than the antigen test (Table 1).

RSV Antigen test: Nasopharyngeal swab specimens were tested using the BinaxNOW™ RSV Card (Abbott, ME, USA). It is a rapid immunoassay employing the immunochromatography principle in a cassette form for the detection of the viral fusion protein. The nasopharyngeal swab was immersed into the assay reagent and then discarded. The mixture of sample and assay reagent was then added to the sample position on the cassette, where it reacted with a conjugate antibody against the RSV fusion protein if present. The result was visualized as a pink to purple color development at the sample line. According the manufacturer, the specificity and sensitivity of the assay are 93% each (BinaxNOW RSV card product instructions leaflet, https://ensur.invmed.com/ensur/broker/ensurbroker.aspx?code=IN430002&cs=26646015, access date: 1 November 2022).

RSV PCR test: The Xpert Xpress Flu/RSV kit (Cephid, CA, USA) was used for the detection of the RSV in nasopharyngeal swab specimens. The assay was run on the GeneXpert instrument (Cephid, CA, USA). It is a reverse transcriptase-based real-time PCR (RT-PCR) assay targeting a nucleocapsid gene fragment of both RSV A and B genotypes. The sensitivity and specificity of the assay are 98.2% and 99.1%, respectively (https://www.cepheid.com/Package%20Insert%20Files/Xpress-Flu-RSV-US-IVD-ENGLISH-Package-Insert-301-7239-Rev.%20D.pdf, access date: 1 November 2022).

Ethical approval: The ethical approval for the study was granted by the Institutional Review Board (IRB) at Imam Abdulrahman Bin Faisal University (IRB-2022-01-127).

Statistical analysis: All data were tabulated in Excel spread sheets. The calculation of frequencies and percentages were performed in Excel. The chi-square and the chi-square for linear trend tests were calculated using the OpenEPI online tool employing the two-by-two table and the dose–response functions, respectively (www.openepi.com, access date: 1 November 2022). The two-tailed *p*-value was used and considered significant if less than 0.05.

## 3. Results

The RSV antigen or nucleic acid were detected in 26.3% (336/1279) of the tested respiratory samples. The RSV positivity was 17.4% using the antigen test and 36.3% using the PCR test. The majority of the study population were Saudis (80.8%), followed by Yemenis (3.2%), Egyptians (1.1), Syrians and Pakistanis (0.8% each), Sudanese and Somalians (0.6% each), Kuwaitis (0.5%), and Emiratis and Filipino (0.4%), with sporadic cases of Indians, Jordanians, Qataris, and Moroccans.

There was no statistically significant difference in the frequency of RSV infection between males and females (*p* 0.021, CI 95% 0.70–1.16) (Table 2).

RSV infection was more prevalent among children of younger ages (below 5 years), and the prevalence decreased among children of older ages. This tendency was statistically significant (*p*-value < 0.001 for chi-square for linear trend) (Table 2).

All cases were suffering from respiratory tract infections with manifestations of bronchiolitis or bronchopneumonia. Two cases had additional bacterial infections, one case had cholesteric jaundice, and one case had asthma. The most common clinical presentations associated with RSV infection were cough, fever, decreased feeding, decreased activity, rhinorrhea, shortness of breath, vomiting, and respiratory distress (Table 3).

Two-thirds of the RSV-positive cases required hospitalization (Table 3 and Table 4). The average hospital stay due to RSV infection was 6.5 days (range 0–56 days). The rate of hospitalization was higher among infants and younger children and decreased with age (*p*-value < 0.001, chi-square for linear trend) (Table 4). Elderly patients above 60 years also required hospitalization, but there was only one case in the study (Table 4). There was no statistically significant difference in the average rate of hospitalization between males and females (mean females 4.9 days, mean males 4.4 days, *p*-value 0.371). No deaths were reported during the period of the study due to RSV infection.

**Figure 1:**
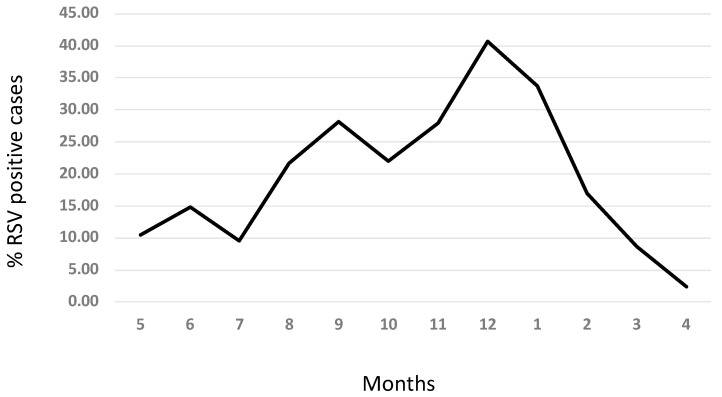
Seasonal pattern of RSV infection.

RSV infection was detected throughout the year from respiratory samples; however, it was more prevalent between August and February and decreased appreciably between March and July (Figure 1). The peak level of infection was during December and January (Figure 1).

**Figure 2:**
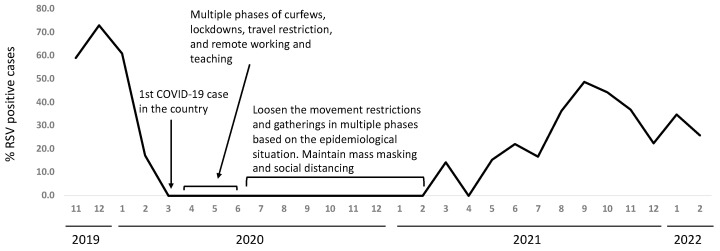
Seasonal pattern of RSV infection during and after the COVID-19 pandemic.

There were no RSV cases reported starting from March 2020 and likewise none during the winter of 2020 and 2021. The cases increased again in August 2021, with an earlier peak than usual (Figure 2). There was a 2-fold increase in the number of RSV-positive cases in the year 2021 (78 cases) compared to the average positive cases from previous years (39.3 cases, range 21–67).

## 4. Discussion

RSV was detected in more than one-quarter of the respiratory infections among patients tested for RSV during the study period. This is similar to the prevalence reported by other studies except for one study from Al-Qassim, where the prevalence was 45% (reviewed in [14]). The PCR assay detected more RSV cases than the antigen assay. It is tempting to assume that the PCR test is more sensitive; however, lack of replica testing of the same sample with both assays in our study precludes such conclusion. Previous studies have indicated that the rapid antigen test for RSV has lower sensitivity than the RT-PCR assay and performs better in younger age groups than in adults [17].

There was no statistically significant difference in the rate of RSV infection among males and females. Some studies have reported such difference from the eastern Mediterranean region [13,18]. However, there was no statistically significant difference in disease severity between males and females, as shown by the average days of hospitalization.

The age prevalence of RSV in our study follows the international and local trend, as it is more prevalent in younger children and decreases with increasing age till the age of above 65 years, where the prevalence of infection increases again [10,13,14]. However, the number of adult patients in the current cohort is limited, and therefore, no firm conclusion can be made about the prevalence of RSV in adults and elderly.

No prophylactic treatment with palivizumab was previously provided to any of the study participants. In 2018, palivizumab was recommended by the Saudi authority to be used for infants based on the gestational age at birth, body weight, and comorbidities (chronic lung disease and congenital heart disease) [19]. No such risk factors were reported in our study population. This also probably explains the absence of deaths among the study population due to RSV infection. However, the high rate of hospitalization due to RSV infection among young children less than 5 years of age and the expected high cost of this hospitalization, as estimated by other studies, indicates the need to provide prophylactic vaccination for this age group.

In the current study, RSV infection was reported throughout the year, with increased prevalence during the winter season and decreased level outside this season, and therefore, the seasonal pattern of RSV infection follows the general seasonal pattern observed in the Northern Hemisphere [4]. A similar pattern was reported by other studies from the region [15,20,21]. Saudi Arabia is located in western Asia to the east of the Red Sea (latitude 23.8859° N, 45.0792° E), occupying most of the Arabian Peninsula.

Interestingly, the RVS cases dropped down to zero, in line with the implementation of lockdown and physical distancing measures in response to the COVID-19 pandemic. Saudi Arabia had one of the most stringent interventions to combat the spread of SARS-CoV-2. These interventions apparently led to the slowdown of the spread of RSV in addition to SARS-CoV-2. There were no RSV cases reported during the winter of 2020–2021. With the relaxation of the COVID-19-control interventions, an out-of-season appearance of RVS cases was observed, with a different peak time of infection. The number of RSV positive cases increased in the year 2021 following the COVID-19 pandemic compared to the average of all previous years. However, this increase in the average RSV cases in 2021 should be carefully interpreted. The RSV-Ag test was more widely used in previous years until 2018. This might have underestimated the prevalence of RSV in previous years, bearing in mind the suggested lower sensitivity of the Ag test compared to the PCR test, which was widely used in 2021. Similar out-of-season appearances of RSV have been reported in different regions from the world [22,23,24,25]. The out-of-season RSV resurgence was justified by the hypothesis that the reduced exposure to the virus lead to reduced immune stimulation to sustain the humoral immune response against the virus and therefore increased the number of susceptible individuals [26,27]. However, a study from Germany measured the anti-RSV antibodies in the population of a small rural town and found that the decrease in the anti-RSV antibodies was minor and could not alone explain the increase in number of cases [28]. They attributed the out-of-season RSV resurgence to rather a time-related pressure that leads to delay in the initial catch-up of infection during the COVID-19 restrictions, which led to the unusual start of the season [28].

Despite the fact that the study hospital is a large reference health center in the region and accepts patients from the whole province, this study implies the inherent limitations of a single-center retrospective study, such as the selection bias and presence of confounding factors. Additionally, the use of two different assays with different sensitivities may have affected the total outcome. Furthermore, the lack of information about other microbial causes of respiratory infection among the study cohort precludes firm conclusion about the magnitude of the role played by RSV in respiratory tract infections.

## 5. Conclusions

Bearing in mind the study limitations, our study showed that RSV infection is possibly one of the important causes of morbidity and hospitalization among children in eastern Saudi Arabia, and it follows a seasonal pattern similar to that observed in the Northern Hemisphere. However, a post-COVID-19 seasonal prevalence pattern should alert the physicians to be aware of RSV infections at out-of-season times. More studies are perhaps required at a later time point to show if the seasonal pattern will revert back to its pattern before the COVID-19 era.

## Figures and Tables

**Table 1:** Annual frequency of RSV testing according to the type of test.

Year	Ag Test	%	RT-PCR Test	%	Total
2015	106	100.0	0	0.0	106
2016	169	100.0	0	0.0	169
2017	136	94.4	8	5.6	144
2018	162	88.5	21	11.5	183
2019	78	40.8	113	59.5	191
2020	16	10.7	134	89.3	150
2021	10	3.9	245	96.1	255
February 2022	2	2.5	79	97.5	81

**Table 2:** The rate of RSV infection according to gender and age group.

	Positive	%	Negative	%	Total	*p*-Value
	336	26.3	943	73.7	1279	
Male	180	25.4	530	74.6	710	0.405
Female	156	27.4	413	72.6	569	
Age group (Years)						
<3	161	36.2	284	63.8	445	<0.001 *
3–5	108	20.6	416	79.4	524	
6–9	63	23.7	203	76.3	266	
10–14	1	4.8	20	95.2	21	
15–60	2	13.3	13	86.7	15	
>60	1	12.5	7	87.5	8	

* *p*-value for linear trend.

**Table 3:** Clinical manifestation and days of hospitalization associated with RSV infection.

Clinical Presentation	No.	%
Cough	205	72.7
Fever	175	62.1
Decrease feeding and activity	103	36.5
Rhinorrhea	102	36.2
Shortness of breath	86	30.5
Vomiting	78	27.7
Respiratory distress	61	21.63
Days of hospitalization		
0	111	33.0
1–3	44	13.1
4–6	87	25.9
7–10	71	21.1
>10	23	6.9

**Table 4:** The frequency of hospitalization due to RSV infection in different age groups.

Age Group	No Hospitalization	%	Required Hospitalization	%	Total	*p*-Value *
<3	28	17.4	133	82.6	161	<0.001
3–5	30	27.8	78	72.2	108	
6–9	50	79.4	13	20.6	63	
10–14	1	100.0	0	0.0	1	
15–60	2	100.0	0	0.0	2	
>60	0	0.0	1	100.0	1	
Total	111	33.1	225	67.3	336	

* *p*-value for linear trend.

## Data Availability

Data are available upon request from the corresponding author.
